# Quality-of-life measurement in randomised controlled trials of mental health interventions for autistic adults: A systematic review

**DOI:** 10.1177/13623613241287586

**Published:** 2024-10-22

**Authors:** Amanda Timmerman, Vasiliki Totsika, Valerie Lye, Laura Crane, Audrey Linden, Elizabeth Pellicano

**Affiliations:** 1 University College London, UK; 2 University of Warwick, UK; 3 The Tavistock and Portman NHS Foundation Trust, UK; 4 Millennium Institute for Care Research (MICARE), Chile; 5 University of Birmingham, UK; 6 The Open University, UK

**Keywords:** anxiety, community involvement, co-production, depression, participatory research

## Abstract

Autistic people are more likely to have co-occurring mental health conditions compared to the general population, and mental health interventions have been identified as a top research priority by autistic people and the wider autism community. Autistic adults have also communicated that quality of life is the outcome that matters most to them in relation to mental health research and that they want to be involved more actively in the research process. Our systematic review aimed to determine the extent and nature of (1) quality of life measurement in randomised controlled trials of mental health interventions for autistic adults and (2) community involvement taking place within identified randomised controlled trials. We searched Medline, Embase, APA PsycInfo, Web of Science and grey literature sources. After screening over 10,000 records, 19 studies were eligible and five of those studies measured quality of life as an outcome. Of those five, three included community involvement and two did not report on community involvement. We conclude there is a need for increased use of quality of life measurement when trialling mental health interventions, including the use of measures validated for autistic adults – which would be facilitated by greater autistic involvement in the research process.

**Lay Abstract:**

Autistic people are more likely to have health problems than the general population. They, and people who care about them, have said mental health research is very important, and some autistic adults have said quality of life is the most helpful area to research when focusing on mental health. Autistic people should also be more deeply involved in making decisions in research. Our review aimed to find out if and how quality of life is being measured when mental health treatments are being tested, and how autistic people and the wider autism community are involved in these studies. We searched four databases and other sources and found over 10,000 records. But just 19 research studies were testing mental health treatments for autistic adults, and only five of those measured quality of life. When they did measure quality of life, it was measured in different ways and there was not much information given on how communities were involved. We suggest mental health research should measure quality of life more often and in ways that are more helpful for autistic people. Our analysis found that mental health research needs to include a wider variety of autistic people, and autistic people should be more involved in the various parts of research.

Systematic reviews have consistently reported a higher prevalence of mental health conditions in the autistic population, particularly among adults, compared to the wider population (Hossain et al., 2020; Lai et al., 2019). Unfortunately, autistic adults experience multiple barriers to accessing and receiving support for their mental health needs (Camm-Crosbie et al., 2019; Crane et al., 2019). Barriers can include individual autistic characteristics, such as communication and sensory differences (Brede et al., 2022). They can also include broader, systemic challenges for autistic people, including barriers accessing primary healthcare (Johnson et al., 2022; Shaw et al., 2023), lack of accommodations in healthcare environments (Doherty et al., 2023), an over-emphasis on neurotypical norms during patient–clinician interactions (Brede et al., 2022), and a lack of understanding of autism across multiple professions (Corden et al., 2021). These barriers persist despite autistic adults – including those legally represented by guardians and those likely to have an intellectual disability – prioritising both a need for better access to healthcare and more research on co-occurring mental health conditions (Gotham et al., 2015).

It is unsurprising that mental health is a top priority in autism research (Cusack & Sterry, 2015; Roche et al., 2020) given that current prevalence estimates for anxiety (27%) and depressive (23%) disorders among autistic adults (Hollocks et al., 2018) far exceed such estimates in the general population; around 7% (Baxter et al., 2013) and 5% (World Health Organization [WHO], 2021), respectively. In a recent *Lancet Commission*, professionals, academics and members of the autistic and autism communities^1^ all signalled an urgent need for action to improve not only mental health interventions and services for autistic people, but also quality of life (QoL) (Lord et al., 2022). The World Health Organisation defines QoL as, ‘an individual’s perception of their position in life in the context of the culture and value systems in which they live, and in relation to their goals, expectations, standards and concerns’ (Skevington et al., 2004). In recent years, autistic adults have ranked QoL as the outcome that matters most to them in relation to mental health research (Benevides et al., 2020). Furthermore, on average, QoL for autistic adults is estimated to be lower than that of the general population (Ayres et al., 2018; Graham Holmes et al., 2020), and poor mental health has been found to be associated with poor QoL for autistic adults (Mason et al., 2018; Sáez-Suanes & Álvarez-Couto, 2022). There is evidence that these issues persist into later adult life, highlighting a need for suitable evidence-based interventions at all stages of adulthood (Mason et al., 2019; Roestorf et al., 2022).

Currently, various psychological and pharmacological interventions are used to help autistic people experiencing mental health problems, including antidepressants, cognitive behavioural therapy and mindfulness-based therapies (Linden et al., 2022). However, for some autistic people the support received is ill-suited to their needs, including through inappropriate use of medication, services based around neurotypical norms, and clinicians reportedly lacking awareness and understanding of autism (Brede et al., 2022). Likewise, mental health professionals themselves have identified numerous challenges in delivering the individualised treatment needed, reporting concerns with a lack of evidence, training and support to guide them (Moore et al., 2023). These issues may stem from a lack of evaluation of benefits and harms for autistic people, as well as high risk of bias, as identified in randomised controlled trials (RCTs) of psychological and pharmacological interventions for anxiety and depression for autistic people (Linden et al., 2022).

Moreover, the extent to which appropriate QoL measurement is taking place when evaluating mental health interventions for autistic adults is unclear, with potential issues identified surrounding community involvement in the development and validation of current measures (Simpson et al., 2024). One reason for this apparent oversight could be related to the privileging of outcomes typically defined by non-autistic researchers and clinicians rather than what has been communicated as meaningful by autistic people themselves (Pellicano et al., 2022). A growing number of researchers and members of the autistic community have suggested that involving community members that are affected by the research in a decision-making capacity could be one crucial way to address this issue (Fletcher-Watson et al., 2019; Pellicano & den Houting, 2021; Pellicano et al., 2022).

A purported benefit of participatory approaches is that the research and its findings should be more relevant to the needs and preferences of community members and more consistent with their values (Callard et al., 2011; Hickey et al., 2018). Until recently, the vast majority of autism research occurred without any input from autistic people, their family members or other supporters (Jivraj et al., 2014). There is, however, a slow but growing movement towards including community members in autism research (den Houting et al., 2021; Pickard et al., 2021; Tan et al., 2024b), owing in large part to the rise in advocacy from autistic activists and those within the broader neurodiversity movement, who have rightly demanded that research needs to have a more meaningful impact on autistic people’s everyday lives (see Fletcher-Watson et al., 2019; Milton, 2014; Nicolaidis, 2019; Pellicano et al., 2022; Pellicano & Stears, 2011, for discussion). Regarding mental health research specifically, there have been studies co-produced between autistic community members and non-autistic researchers on mental health experiences of autistic young adults including support received (Crane et al., 2019), on the adaptation of psychological therapies for autistic people (Stark et al., 2021), as well as studies generating priorities for mental health research for autistic adults (Benevides et al., 2020). Little is known, however, about the nature and extent of community involvement in relation to RCTs of mental health interventions, despite RCTs being the primary and most accepted research design through which the efficacy of interventions is tested (Schulz et al., 2010).

## The current study

Given the importance of QoL in relation to the mental health of autistic adults, the lack of clarity surrounding its measurement, and calls for increased community involvement throughout the research process, the current systematic review sought to address the following research questions:

1. How frequently is QoL measured in RCTs of mental health interventions for autistic adults?
2. How is QoL being measured in RCTs of mental health interventions for autistic adults?
3. What role does community involvement play in the extent and nature of QoL measurement in RCTs of mental health interventions for autistic adults?

## Method

We followed the Preferred Reporting Items for Systematic Reviews and Meta-Analyses (PRISMA) guidelines (Page et al., 2021), and the review protocol was registered with PROSPERO prospectively; registration number CRD42022340298.

### Review criteria

Studies eligible for inclusion were RCTs with participants aged 18 and above with a formal autism diagnosis. For the purposes of this review, ‘mental health intervention’ was defined as a pharmacological, non-pharmacological, or combined pharmacological and non-pharmacological intervention aiming to prevent, treat or manage mental health problems, which also used standardised outcome measures specific to mental health problems. There were no constraints on the type of mental health problems being considered.

To maximise the inclusiveness of our approach, we included studies whose autistic participants were reported as having an intellectual disability (ID) and/or having an IQ < 70. For these studies, behaviour problems (e.g. self-injurious behaviour, aggression, and irritability) were considered indicators of mental health problems (following Painter et al., 2018, and Westlake et al., 2021). Therefore, RCTs trialling interventions targeting behaviour for this group were considered eligible for inclusion provided other criteria were met. In addition, unpublished and non-peer-reviewed studies (e.g. preprints) were eligible for inclusion provided other inclusion criteria were met. There were no restrictions on publication period.

We excluded the following studies from review: (1) non-English language papers, (2) studies where the full text could not be retrieved, (3) reviews (including systematic reviews), conference proceedings, opinion pieces, and study designs other than RCTs; and (4) studies with a mixture of child and adult participants, if data from autistic adults could not be isolated for extraction.

### Search strategy

The search strategy was created collaboratively by the research team, with guidance from an information scientist. A combination of keywords and Medical Subject Headings (MeSH) terms specific to each bibliographic database were utilised as well as RCT search filters to ensure adequate sensitivity.

Medline, Embase and APA PsycInfo were searched via Ovid in June 2022. Web of Science (WoS) was also searched in June 2022, using keywords only because MeSH terms are not included in WoS search functionality. In addition, the following grey literature sources were searched in June 2022: APA PsycExtra (via Ovid); ClinicalTrials.gov; and WHO International Clinical Trials Registry Platform (ICTRP). The clinical trial registries were searched for autism intervention studies at phases two (efficacy testing) to four (post-approval/post-marketing trials). All searches were repeated on 7 August 2022 and again on 25 May 2023 (see supplementary materials for all search strategies).

Following study selection, forward searching took place using the Web of Science ‘Times Cited’ function to screen the citations of eligible studies. Backward citation searching was also performed using the reference lists of all eligible studies. Where clinical trials had a subsequent journal publication, the journal publication was used as the eligible paper for analysis, superseding the trial record.

### Study selection

EndNote was used for deduplication of the search results. Separate screening documents for first-stage screening of titles and abstracts and second-stage screening of full-text were produced (see supplementary materials). These documents detailed the inclusion and exclusion criteria and were made available to reviewers.

For first-stage screening, one reviewer screened all search results, recording decisions via sorting potential studies for inclusion into a separate EndNote group folder. A second reviewer independently screened 10% of the search results (randomly selected) using the same method. For second-stage screening, one reviewer screened all remaining studies for potential inclusion, recording decisions via the screening document with supporting comments. A second reviewer independently screened 20% of the included studies (randomly selected) using the same method.

Inter-rater reliability was calculated using STATA, resulting in a Cohen’s kappa of 0.66 and inter-rater agreement of 99.35% at first-stage screening, and a Cohen’s kappa of 0.69 and inter-rater agreement of 85.71% at second-stage screening. Disagreements were resolved through discussion and, when necessary, a third reviewer was consulted to reach consensus.

### Data synthesis

A bespoke Excel data extraction template was created for the purposes of the review. The following data was extracted: authors, publication year, title, country, study aims, study design intervention name, intervention type, mental health outcome measures, recruitment methods, data collection methods, total sample size, participant age, sex/gender, ethnicity, intellectual disability/IQ < 70, diagnoses, comparison group, socioeconomic status (SES), QoL measures used and rationale, QoL results (if applicable), and community involvement.

One reviewer extracted data from all included papers while a second reviewer independently extracted data from 20% of the included papers (an oversight meant that this figure was higher than the 10% stated in the protocol). Inter-rater reliability was calculated as a percentage of whether there was agreement between raters on the data extracted, with a resulting agreement of 75%. Cohen’s kappa could not be calculated at this stage owing to the nature of the data being rich and textual rather than binary responses. Disagreements were resolved through discussion.

A classification checklist was created based on the QoL measures identified in systematic reviews by Ayres et al. (2018), Haraldstad et al. (2019) and Pequeno et al. (2020) (see supplementary materials). These reviews identified all QoL measures available, and we used their findings to classify our measures. To assess how frequently QoL was measured in RCTs of mental health interventions for autistic adults (research question one), we counted the number of studies that included a QoL measure. Studies that included QoL measures were then analysed narratively in terms of rationale for chosen measure, validity of the measure and QoL outcomes (research question two). Narrative synthesis was used to analyse the level of community involvement and any other forms of research co-production in the eligible studies (research question three).

### Quality appraisal of eligible studies

The Critical Appraisal Skills Programme (CASP, n.d.) Randomised Controlled Trial Standard Checklist was used for quality assessment of all eligible studies. The tool includes four sections: Section A, screening questions concerning study validity as an RCT; Section B, questions regarding how methodologically sound the study is; Section C, questions surrounding what the results were, how they are reported and the cost-effectiveness of the intervention; and Section D, appraising whether the findings can help locally. The use of a scoring system is not recommended by CASP, therefore numerical scoring is not included in the results.

One reviewer quality appraised all eligible studies, and a second reviewer independently appraised 20% of eligible studies. Inter-rater reliability was calculated using STATA, yielding an inter-rater agreement of 87.69%, and Cohen’s kappa of 0.81. Disagreements were resolved through discussion.

### Community involvement statement

Our research team includes both autistic and non-autistic researchers who contributed to developing the research questions, study design, implementing measures, gathering data, analysing results, interpreting findings and disseminating the research.

## Results

### Search results

**Figure 1.**
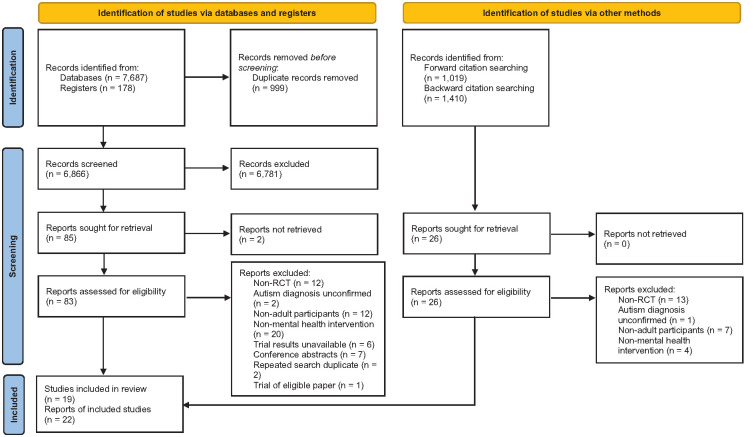
PRISMA flowchart showing search process and study selection.

Of the 10,294 unique records screened from databases, registers and backward and forward citation searching, 22 reports (from 19 studies) met the inclusion criteria (see Figure 1).

### Study characteristics

Table 1 provides a summary of the 19 included studies. The Braden et al. (2022) and Pagni et al. (2020) reports were from the same study. Similarly, the Horwood et al. (2021), Russell et al. (2019) and Russell et al. (2020) reports were all from the same study. Most reports were from USA-based studies (*n* = 8, 36%), followed by studies based in the UK (*n* = 7, 32%), Netherlands (n = 3, 14%), Sweden (n = 2, 9%), Korea (n = 1, 5%) and Taiwan (n = 1, 5%). Most interventions were non-pharmacological in nature (n = 19, 86%) with the remainder being pharmacological (n = 2, 9%) or a combination of pharmacological and non-pharmacological (n = 1, 5%). Anxiety and/or depression were the mental health outcomes of focus (n = 15, 68%), though some studies used measures assessing multiple mental health problems (n = 5, 23%).

**Table 1.** Summary characteristics of eligible RCT reports.

Study (Country)	Mental health interventions^a^	Mental health outcomes^a^	Sample, *n*	Age, Mean(*SD*)^b^	Gender M: F^c^	Sex M: F^c^	Reported ethnicity	ID or IQ < 70?	SES indicators reported^d^
Braden et al. (2022) **(USA)** ^e,f^	Mindfulness-based Stress Reduction (MBSR) & active control intervention	Anxiety, depression (self-report)	70	31.4 (13.3)	—	34:21	*n* = 47 White = 91% Asian = 4% Hispanic = 2% African American = 2%	No	Education
Chien et al. (2021) **(Taiwan)**	Programme for the Education and Enrichment of Relational Skills (PEERS^®^)	Anxiety (self-report)	82	26.5 (5.4)	69:13	—	—	No	Education, living situation, employment
Coulter et al. (2022) **(UK)**	Heart Rate Variability Biofeedback (HRV)	Anxiety, depression (self-report)	20	—	16:4	—	—	No	Education, employment
Danforth et al. (2018) **(USA)**	3,4-methylenedioxy-methamphetamine (MDMA) with adapted mindfulness-based DBT	Anxiety (clinician-administered) Depression (self-report)	12	31.3 (8.8)	—	10:2	White/Caucasian = 50% Latino/Hispanic = 17% Asian/Pacific Islander = 8% Middle Eastern = 8% Asian & Caucasian = 8% Hispanic & Caucasian = 8%	—	Employment
Gaigg et al. (2020) **(UK)**	Mindfulness-based course (MBT) & Cognitive Behaviour Therapy (CBT)	Anxiety, depression (self-report)	54	43.3 (12.2)	43:11	—	—	No	—
Hesselmark et al. (2014) **(Sweden)**	Cognitive Behavioural Therapy (CBT) & Recreational Activity (RA)	Multiple (including CMDs) (self-report)	75	31.8 (9.0)	41:34	—	—	No	Employment, living condition, education
Horwood et al. (2021) **(UK)** ^g^	Guided Self-Help (GSH)	Depression (2 scales self-report, 1 scale clinician-administered)	21	39.2 (11.7)	17:4	—	White British = 100%	—	Employment, deprivation decile
Lee et al. (2022) **(USA)**	Puzzle Walk & GoogleFit (mobile apps)	Anxiety (self-report)	24	29.5 (9.7)	—	9:15	—	No	Education, employment
McDougle et al. (1998) **(USA)**	Risperidone	Self-injurious behaviour (informant-report)	31	28.1 (7.3)	—	22:9	White = 77% African Americans = 19% Hispanic = 3%	Mixed	—
McVey et al. (2016) **(USA)**	PEERS for Young Adults (PEERS-YA)	Anxiety (self-report)	47	20.2 (2.7)	—	38:9	Non-Hispanic = 96%	No	Household income, parent education
Oh et al. (2021) **(USA)**	PEERS for Young Adults (PEERS-YA)	Anxiety, depression (self-report)	37	23.4 (3.8)	36:1 (unclear)	—	Korean = 100%	No	Education
Oswald et al. (2018) **(Korea)**	Acquiring Career, Coping, Executive control, Social Skills (ACCESS) Programme	Multiple (including CMDs) (self-report)	44	25.1 (6.4)	31:13 (unclear)	—	Caucasian = 73.2%	No	Living independently
Pagni et al. (2020) **(USA)** ^e^	Mindfulness-based Stress Reduction (MBSR)	Anxiety, depression (self-report)	28	31.8 (13.0)	—	19:9	—	No	—
Pagni et al. (2023) **(USA)**	Mindfulness-based Stress Reduction (MBSR)	Anxiety, depression (self-report)	78	32.7(13.4)	—	49:29	—	No	—
Pahnke et al. (2022) **(Sweden)**	adapted acceptance and commitment therapy (NeuroACT)	Stress, anxiety, depression (self-report)	39	39.1(12.2)	21:18	—	—	No	Education, Occupation
Quadt et al. (2021) **(USA)**	Aligning Dimensions of Interoceptive Experience (ADIE)	Anxiety, depression (self-report)	121	30 (—)	57:58	55:66	—	No	Education
Russell et al. (2019) **(UK)** ^g^	Guided Self-Help (GSH)	Anxiety, depression, OCD (4 scales self-report, 1 scale clinician-administered)	70	37.8 (13.2)	51:18	—	*n* = 69 White = 96%	—	Living situation, education, employment, financial stress
Russell et al. (2020) **(UK)** ^g^	Guided Self-Help (GSH)	Anxiety, depression, OCD (4 scales self-report, 1 scale clinician-administered)	70	37.8 (13.2)	51:18	—	*n* = 69 White = 96%	—	Living situation, education, employment, financial stress
Spek et al. (2013) **(Netherlands)**	Mindfulness-based-therapy for Autism Spectrum Disorders (MBT-AS)	Multiple (including CMDs) (self-report)	41	42.2 (11.1)	27:14	—	—	No	—
Strydom et al. (2020) **(UK)**	Positive Behaviour Support (PBS)	Multiple (including CMDs) (informant-report)	113	34.6 (14.0)	83:30	—	White = 66%	Yes	Living situation
Wijker et al. (2020) **(Netherlands)**	Animal Assisted Therapy (AAT)	Multiple (including CMDs) (self-report)	53	—	29:24	—	—	No	—
Willemsen-Swinkels et al. (1995) **(Netherlands)**	Naltrexone Hydrochloride	behaviour that challenges (informant-report)	33	29 (6)	—	27:6	—	Yes	Living situation

Note: CMDs – common mental disorders, primarily depressive and anxiety disorders; DBT – Dialectical Behavioural Therapy; ID – Intellectual Disability; IQ – Intelligence Quotient; OCD – obsessive compulsive disorder; SES – socioeconomic status. Cells marked ‘—’ indicate information not reported. Supplementary materials were consulted.

a Where autistic adults had co-occurring ID, behavioural problems were considered indicators of mental health problems, making behavioural interventions eligible for review.

b Several studies did not report age to at least one decimal place. Where possible, study authors were contacted to provide missing information.

c Due to missing data, not all numbers for M: F add up to total *n* of sample. For this reason, ratios have not been simplified. Two papers were unclear in whether they were reporting sex or gender.

d SES indicators varied across papers to include numerical data, but data summarised in the main text. Terms have been unified to facilitate reporting.

e Braden et al. (2022) and Pagni et al. (2020) are papers reporting on the same study.

f Braden et al. (2022) reported on two studies. Data included are from Study 2 only as Study 1 was not an RCT.

g Horwood et al. (2021) and Russell et al. (2019, 2020) are papers reporting on the same study.

Of studies that reported age and standard deviation, a total of 871 autistic adults participated with a mean age of 32.3 years (*SD* = 11.7). Papers varied in their reporting of ‘sex’ versus ‘gender’, with two reports being unclear regarding which they were reporting. However, of the 661 participants whose gender was clearly reported, most were men (*n* = 437, 66%). Likewise, of the 401 participants whose sex was clearly reported, most were male (n = 224, 61%). Quadt et al. (2021) reported sex assigned at birth as well as gender, which included six participants whose gender was reported as ‘other’.

Of the 400 participants whose ethnicity was reported (across eight studies), most were reported as being white or ‘Caucasian’ (*n* = 246, 62%). One paper used this term when reporting on race as a demographic characteristic (McVey et al., 2016), but reported on ethnicity only in terms of ‘non-Hispanic’.

Most papers (*n* = 15, 68%) reported excluding autistic adults with co-occurring ID or those whose IQ was less than 70. Two further studies likely excluded autistic adults with ID despite not stating this explicitly. Specifically, Danforth et al. (2018) required participants to have at least two years of college education and Russell et al. (2019) required participants to be able to understand study materials, however one of Russell’s four recruitment pathways (Adult Autism Spectrum Cohort – UK at Newcastle University) constrained their participant database search by ID. Only two studies clearly included participants with ID: McDougle et al. (1998) and Strydom et al. (2020).

Most papers (*n* = 16, 73%) reported one or more indicators of SES such as education level, employment status, income and living situation, but there was large heterogeneity in the SES data that were reported and how they were reported. Occupation was reported most consistently. Across the six studies that reported data on occupation, 302 autistic adults were represented with 111 (37%) reported as being in some form of employment or training (including unpaid/voluntary workers and students).

### Quality appraisal

More than half the 19 RCTs (*n* = 11, 58%) were statistically underpowered and identified themselves as pilot, preliminary, feasibility, or exploratory studies. The nature of four RCTs was unclear either because the authors neither specified the type of RCT nor provided information that the RCT was designed following a power analysis (n = 4, 21%).

Just over half of the 22 papers were rated as being of medium quality (*n* = 12, 55%), followed by those rated as low quality (n = 8, 36%). Concerns surrounding risk of bias were identified across multiple reports in terms of how randomisation was handled (n = 8, 36%), differences between the intervention and control groups (n = 13, 59%) and how blinding was managed (n = 20, 90%). The latter concern seemed due in part to the inherent difficulty of blinding non-pharmacological interventions. However, for half of the papers, information regarding blinding was unclear (n = 11, 50%).

Though no reports satisfied all CASP criteria, one pilot study (Braden et al., 2022) was rated as high quality due to the level of effort employed to blind a non-pharmacological intervention while satisfying most other CASP criteria, particularly comprehensive reporting of the intervention’s effects including power calculations and effect estimates. The remaining CASP criteria, which Braden et al. (2022) did not satisfy, contained considerations typically not expected of preliminary studies. Furthermore, one fully powered study, Pagni et al. (2023) was rated as high quality due to the robust study design that incorporated the use of an active control intervention, as well as satisfying the majority of CASP criteria.

For most reports (*n* = 18, 82%), it was difficult to determine whether the benefits of the interventions outweighed the potential harms or cost, due to non-reporting of effect sizes, little or no information regarding potential harms or adverse outcomes, and no cost-effectiveness analyses.

Regarding generalisability, none of the interventions trialled could be applied to the local context due low/unclear ethnic diversity or under-representation of autistic people with ID. Finally, for many reports, it was difficult to determine if the interventions would provide greater value to autistic people compared to other intervention options (*n* = 19, 86%) as there was little to no information provided regarding resourcing e.g. time, finance, skill development or training for the interventions. (See supplementary materials for CASP results table.)

### Research Question 1: how frequently is QoL measured in RCTs of mental health interventions for autistic adults?

Of the 19 eligible RCTs of mental health interventions, five (26%) measured QoL using at least one QoL measure (see Table 2).

**Table 2.** Quality of life measures in included studies.

QoL measure^a^	Braden et al. (2022) ^b^	Hesselmark et al. (2014)	Pahnke et al. (2022)	Russell et al. (2019, 2020)^c^	Strydom et al. (2020)
**EQ-5D**	—	—	—	Yes	Yes
**QOLI**	—	Yes	Yes	—	—
**SF-12**	—	—	—	Yes	—
**SWLS**	—	—	Yes	—	—
**WHOQOL-BREF**	Yes	—	—	—	—
**Reported RCT Type**	Pilot	Preliminary	Pilot	Feasibility	Powered
**Summary of QoL Results:**	Mental health-related QoL for both the MBSR and active control groups improved. MBSR had greater improvement in disability-related QoL than the active control. Women in both groups improved more than men in physical and psychological QoL	QoL for both CBT and RA groups improved post-treatment. No significant difference in effect size between groups.	SWLS: a statistically significant interaction effect, with moderate effect size, in favour of the NeuroACT group compared with TAU QOLI: Not statistically significant	EQ-5D-5 L: QoL for GSH intervention group was higher at 16 weeks and 24 weeks compared to TAU. SF-12: normalised physical function appeared to decline for TAU.	EQ-5D-Y: QoL effect size not reported because QoL measure used only for QALYs estimates. PBS found to be cost-effective in terms of QALYs.

Note. CBT – Cognitive Behavioural Therapy; GSH – Guided Self Help; MBSR – mindfulness-based stress reduction; NeuroACT – adapted acceptance and commitment therapy; PBS – Positive Behavioural Support; QALYs – Quality-Adjusted Life Years; QoL – Quality of Life; RA – Recreational Activity; TAU – Treatment as Usual.

a EQ-5D – EuroQol-5 Dimensions (EuroQol, n.d.); QOLI – Quality of Life Inventory (Frisch, 2014); SF-12 – Short-Form Health Survey, 12-item (Ware et al., 1996); SWLS – Satisfaction With Life Scale (Diener et al., 1985); (WHOQOL-BREF – World Health Organisation (WHO) Quality of Life – Brief Version (WHO, 1996).

b Braden et al. (2022) reported on two studies. Data included are from Study 2 only as Study 1 was not an RCT.

c Russell et al. (2019) and Russell et al. (2020) are reports on the same study.

### Research Question 2: how is QoL being measured in RCTs of mental health interventions for autistic adults?

Five different QoL measures that were used across the five aforementioned studies were self-report except for Strydom et al. (2020), where the measure was completed by family or paid carers as proxies.

#### EuroQol-5 Dimensions (EQ-5D)

Two studies used the EQ-5D. The EQ-5D is a group of three health-related QoL measures: EQ-5D-5 L, EQ-5D-3 L and EQ-5D-Y. These tools focus on the five daily dimensions of mobility, self-care, usual activities, pain/discomfort and anxiety/depression (EuroQol, n.d.). The EQ-5D-5 L evaluates the dimensions on five levels of severity whereas the EQ-5D-3 L measures QoL in terms of three levels of severity and both have been validated across multiple patient groups (Janssen et al., 2013). Similarly, the EQ-5D-Y also measures QoL on three levels of severity but has been adapted to be more suitable for youth (children aged 8 – 11 and adolescents aged 12 – 18) and has been validated in several countries (Ravens-Sieberer et al., 2010).

Russell et al. (2019, 2020) used the EQ-5D-5L self-report measure because it had ‘been found to be reliable and valid in the typically developing population’ (Russell et al., 2019, p. 14). According to a recent systematic review, the EQ-5D-5L has not been validated for autistic adults specifically (Feng et al., 2021).

Strydom et al. (2020) used the EQ-5D-Y. They reported that this measure was chosen in accordance with published guidance for the measurement of quality-adjusted life years (QALYs) (Hunter et al., 2015). According to a recent systematic review, the EQ-5D-Y has not been validated for autistic youth specifically (Golicki & Młyńczak, 2022). However, the participants in the Strydom et al. (2020) study were autistic adults with ID and the measure was completed by family members and paid carers as proxies. There appear to be no studies validating the EQ-5D-Y with autistic adults or those with co-occurring ID.

#### Quality of Life Inventory (QOLI)

Two studies used the QOLI. The QOLI is a 32-item, self-report measure of satisfaction with 16 domains: health, self-esteem, goals-and-values, money, work, play, learning, creativity, helping, love, friends, children, relatives, home, neighbourhood and community (Frisch, 2014). Since the Ayres et al. (2018) systematic review on QoL for autistic adults, no study appears to have validated the QOLI with autistic people. No rationale for this choice of measure was reported by Hesselmark et al. (2014). Pahnke et al. (2022) cited satisfactory to good internal consistency and test–retest reliability as their reason for this choice of measure and went on to reported good internal consistency with their own sample.

#### Medical Outcomes Study Short-Form Health Survey (SF-12)

One study used the SF-12 – a 12-item measure of health-related QoL with a focus on the eight domains of physical functioning, role physical, bodily pain, general health, vitality, social functioning, role emotional and mental health (Ware et al., 1996). Khanna et al. (2015) investigated the psychometric properties of version 2 of the SF-12 (SF-12v2) for autistic adults and found acceptable factorial and convergent validity. There appear to be no studies assessing the validity of the SF-12 with autistic people.

Russell et al. (2019, 2020) chose the SF-12 as it had been demonstrated to be reliable and valid for use with people with severe mental health problems. This reason may be why version two of the SF-12 (SF-12v2) was not used, even though it was available as early as 2011 (Montazeri et al., 2011). Moreover, data were only collected for the subscales of normalised physical function and normalised mental health. However, the SF-12 was used in addition to the full EQ-5D-5 L measure in their studies.

#### Satisfaction With Life Scale (SWLS)

Only one study used the SWLS, a five-item scale that measures QoL in terms of: closeness to ideal life, conditions of life, life satisfaction, important things in life and whether one would change their life (Diener et al., 1985). Pahnke et al. (2022) cited satisfactory convergent validity and good internal consistency as the reason for selecting this measure and went on to report satisfactory internal consistency with their sample. There appear to be no studies validating the SWLS with autistic adults.

#### World Health Organisation Quality of Life – Brief Version (WHOQOL- BREF)

Only one study used the WHOQOL-BREF, a 26-item scale that measures QoL in terms of four domains: physical health, psychological health, social relationships and environment (WHO, 1996). The WHOQOL-BREF has been validated for autistic people in a study by McConachie et al. (2018), which also went on to develop and validate a set a set of nine additional autism-specific items (ASQoL) for use alongside the WHOQOL-BREF.

Braden et al. (2022)^2^ reported on two studies, with Study Two being the RCT of interest for this review. They reported choosing the WHOQOL-BREF over the Medical Outcomes Study Short-Form (SF-36) due to the option of using the additional ASQoL items.

### Research Question 3: what role does community involvement play in the extent and nature of QoL measurement in RCTs of mental health interventions for autistic adults?

Of the five studies measuring QoL, only Russell et al. (2019, 2020) reported autistic community involvement (*n* = 1, 20%). The remaining studies did not report on community involvement (n = 4, 80%). Table 3 shows a summary of community involvement of those studies, including where authors provided additional information, detailed below.

**Table 3.** Summary checklist of community involvement.

Involvement	Braden et al. (2022)	Hesselmark et al. (2014)	Pahnke et al. (2022)	Russell et al. (2019, 2020)^a^	Strydom et al. (2020)
Autistic community	Yes	Not reported	Not reported	Yes	—
Autism community	Yes	Not reported	Not reported	—	Yes
Other stakeholder involvement	Yes	Not reported	Not reported	—	Yes

a Russell et al. (2019) and Russell et al. (2020) report on the same study.

#### Braden et al. (2022)

Braden et al. (2022) did not report on community involvement in their paper. Upon contact, however, they recounted that two autistic graduate students contributed to the implementation of these studies and one community autism centre leader was involved in the development of the research questions and design. No further information was provided.

#### Hesselmark et al. (2014) and Pahnke et al. (2022)

No information on community involvement was included in these reports. The first study authors were contacted but no response was received.

#### Russell et al. (2019, 2020)

Two volunteer autistic adults were reported to have helped inform the Guided Self-Help (GSH) intervention session materials of the Russell et al. (2019, 2020) study. The volunteers were reportedly recruited via a newsletter distributed through a service network, and after establishing that their mental health difficulties were not impacting on aspects of their functioning at the time of the study, they were invited to provide feedback in relation to improving the accessibility and content of research materials. They also were able to contribute their skills and lived experience of past mental health support to the research process and suggest changes to any aspects of the guided self-help intervention they felt were important. This took the form of one-to-one meetings between each volunteer and a member of the research team, and volunteers were reimbursed for their time according to INVOLVE guidance (NIHR INVOLVE, n.d.).

Initial session materials were reviewed in the first meeting and the autistic volunteers were able to provide and discuss feedback as well as suggest improvements. This input was provided in an open way without a predetermined agenda. Examples of community-informed change were reported, including: improvements made to the intervention materials’ examples and prompts; changes to the visual layout and format of session materials; and helping to increase suitability and specificity of the intervention’s homework tasks. These tasks took place during the development phase of the GSH, prior to the intervention’s RCT.

#### Strydom et al. (2020)

Strydom et al. (2020) did not report on community involvement in their paper, but upon contact, one author stated that their study primarily involved adults with ID and the Strydom et al. (2020) paper was a secondary analysis of data related to autistic participants with co-occurring ID. An advisory group contributed to the research process and patient involvement took place, though this was not specific to autistic people.

## Discussion

In this systematic review, we aimed to investigate the extent of QoL measurement in mental health interventions for autistic adults – an outcome identified as important by the autistic and autism communities. Strikingly, we found only one quarter of eligible mental health RCTs measured QoL outcomes among autistic adults. Of the five studies that reported using QoL outcome measures, two measured health-related QoL (HRQoL) only, rather than the broader construct of QoL, and two alone provided a clear rationale for their choice of QoL measure in relation to appropriateness for their participants and their study aims.

HRQoL is a dominant measurement approach because of cost-effectiveness considerations. Specifically, health economists aim to evaluate the balance between cost of an intervention and improvements in QoL, and these improvements have only been quantified for dimensions of QoL that are relevant to health (e.g. mobility, pain). However, not all interventions (especially psychosocial ones) targeting mental health problems will be relevant to HRQoL and economists are beginning to realise that mental health interventions require different QoL measures (Keetharuth et al., 2021). There is also increasing recognition that existing HRQoL measures are not suitable for evaluating the benefits of interventions targeted towards neurodivergent populations (Lamsal & Zwicker, 2017).

Furthermore, non-health domains such as relationships, sense of belonging, acceptance in society and autonomy may prove important to an autistic person’s mental health as has been found for members of the general population seeking treatment (Connell et al., 2014). For example, Cage et al. (2018) found that greater autism acceptance predicted lower depressive symptoms for some autistic adults. Further, some autistic adults reported that the support of family and friends was helpful in managing low mood and depression (Jordan et al., 2020). These factors have been highlighted as missing issues when considering QoL for autistic people (McConachie et al., 2020). Unless researched further, in partnership with autistic people, this picture remains incomplete.

Of the QoL measures used, only one had been validated for use with autistic adults; the WHOQOL-BREF with additional ASQoL autism-specific items (McConachie et al., 2018). However, researchers have noted that care must be taken when interpreting social data from this measure (Mason et al., 2022), and further revision, including of the ASQoL addition, is likely necessary to address sex and gender bias (Williams & Gotham, 2021). In addition, there is a lack of clarity surrounding whether findings from studies on the WHOQOL-BREF can be broadly applied to other QoL measures used in the field, with indications that more validation and adaptation may be needed to ensure measures are accessible for autistic people and cover important autism-related factors (Ayres et al. 2018; McConachie et al., 2020; Simpson et al., 2024). Notably, and of particular relevance to this study, some authors have suggested that the WHOLQOL-BREF is not appropriate for measuring the impact of mental health difficulties on the QoL of autistic people (Mason et al., 2022).

More broadly, QoL as a construct – just like wellbeing – needs careful consideration to ensure that autistic people are being engaged ethically and meaningfully in research, as literature suggests normative ideals are being applied to autistic people in ways that potentially erase their personhood and agency (Lam et al., 2021). Further research on the psychometric properties of the tools used should be a priority, as such work would help reduce uncertainty surrounding past findings while justifying the use of available measures in future research, or if necessary, spur the development of novel measures.

Further examination of the studies included in this review highlighted how most excluded autistic adults with ID, despite the high prevalence of ID in the autistic population compared to the general population (estimated to be 29.4%; Rydzewska et al., 2018). The one study measuring QoL for autistic adults with ID (Strydom et al., 2020) utilised the EQ-5D-Y. The EQ-5D has multiple ‘modes of administration’ available including self-report and proxy report (EuroQol, n.d.). The proxy version appears not to have been used in this study despite evidence of limited reliability and validity of the self-report version of the EQ-5D-3L with adults with ID (Russell et al., 2018). This finding highlights a need for the assessment of the psychometric properties of existing measures for autistic people with co-occurring ID. Alternatively, new measures may need to be developed and validated (Ayres et al., 2018).

Regarding research community involvement, only one of the five studies measuring QoL (Russell et al., 2019, 2020) included information regarding precisely how the autistic community was involved in the research process in terms of recruitment, time spent working on the research, and the ways in which community members contributed to intervention design and the research process in terms of skills and experience. The study authors who responded to requests for more information (Braden et al., 2022; Strydom et al., 2020) provided further details on the community involvement that took place in their studies. In both cases, however, involvement was limited. This finding signals a need for more comprehensive reporting of community involvement (Tan et al., 2024a), its influence on outcome selection in autism research, and its capacity to help reach autistic people from underrepresented groups to participate in RCTs – another limitation of the studies included in this review. Measures such as the GRIPP-2 (Staniszewska et al., 2017) have been developed to guide researchers on how to report public and patient involvement in their RCTs, and these measures should be considered for future work in this field.

Regarding study quality, risk of bias appears to be a major issue for mental health intervention research for autistic adults, where it was common for us to find poor randomisation practices and lack of blinding at various levels (see Braden et al., 2022, for an exception), or insufficient reporting of methodology in these areas. A number of studies failed to report or discuss adverse outcomes or potential harms of the interventions – part of a pervasive problem in autism intervention research (Bottema-Beutel et al., 2021a, Crowley, et al., 2021) – making it difficult to determine whether the benefits of the interventions would outweigh the cost for autistic adults in need of mental health support. Likewise, the effects of the interventions being trialled were not compared to the effect estimates of other available mental health interventions, a consideration in the CASP. However, we acknowledge that most studies were preliminary and statistically underpowered, and it would have been difficult for researchers to draw reliable comparisons at this stage.

### Strengths and limitations

Our search strategy was robust having gone through multiple revisions, consulting PRISMA guidance, consultation with a university librarian, incorporating the use of published RCT filters and searching four bibliographic databases with the addition of three grey literature sources. Although we were only able to include papers published in English, research indicates this omission likely would have had little impact on the conclusions of this review (Dobrescu et al., 2021).

Regarding quality appraisal, the use of the CASP allowed the consideration of aspects of study quality of key importance to autism research, such as external validity issues, but its items are a poor match for pilot or preliminary RCTs.

Finally, we did not conduct a meta-analysis due to the nature of our review and the expected high heterogeneity of interventions trialled and outcome measures used across eligible studies. However, as the research pool increases, meta-analyses may be possible for future systematic reviews of mental health interventions for autistic adults.

## Conclusion

In sum, of the 19 RCTs identified trialling mental health interventions, only 5 measured QoL, an outcome valued by autistic people themselves. While autism mental health intervention research is moving towards measuring more meaningful outcomes for autistic people, the results of this review suggest there is much work still to do. These efforts include selecting QoL measures evidenced as appropriate for the autistic population and ensuring community involvement is not only present but transparent. We hope this systematic review will serve to inform future research on mental health interventions for autistic adults, contribute to improved QoL measurement and ultimately improve mental health and QoL for autistic people.

## Supplemental Material

sj-docx-1-aut-10.1177_13623613241287586 – Supplemental material for Quality-of-life measurement in randomised controlled trials of mental health interventions for autistic adults: A systematic review Supplemental material, sj-docx-1-aut-10.1177_13623613241287586 for Quality-of-life measurement in randomised controlled trials of mental health interventions for autistic adults: A systematic review by Amanda Timmerman, Vasiliki Totsika, Valerie Lye, Laura Crane, Audrey Linden and Elizabeth Pellicano in Autism

sj-docx-2-aut-10.1177_13623613241287586 – Supplemental material for Quality-of-life measurement in randomised controlled trials of mental health interventions for autistic adults: A systematic review Supplemental material, sj-docx-2-aut-10.1177_13623613241287586 for Quality-of-life measurement in randomised controlled trials of mental health interventions for autistic adults: A systematic review by Amanda Timmerman, Vasiliki Totsika, Valerie Lye, Laura Crane, Audrey Linden and Elizabeth Pellicano in Autism

## Notes

1. This review uses the term ‘autistic community’ to refer to autistic people of all ages and the term ‘autism community’ to refer collectively to autistic people along with other key stakeholders in autism research such as parents of autistic children, practitioners and policy makers. This approach is in accordance with publication guidance (Autism, n.d.) while also acknowledging the distinction is connected to the sensitive issue of identity-first versus person-first language preference (Bottema-Beutel, et al., 2021b; Kenny et al., 2016; Lei et al., 2021).

2. Braden et al. (2022) also used the World Health Organisation Disability Assessment Schedule (WHODAS); however, this is not typically considered to be a quality of life measure so was not defined as such in this review (Üstün & WHO, 2010).
