# Supplementary material for: Quality-of-life measurement in randomised controlled trials of mental health interventions for autistic adults: A systematic review
Source: Autism. 2024 Oct 22;29(3):579–95. doi: 10.1177/13623613241287586 (PMC11894889; doi:10.1177/13623613241287586)
Supplement: sj-docx-2-aut-10.1177_13623613241287586 – Supplemental material for Quality-of-life measurement in randomised controlled trials of mental health interventions for autistic adults: A systematic review [file sj-docx-2-aut-10.1177_13623613241287586.docx]

# **Quality of life measurement in randomised controlled trials of mental health interventions for autistic adults: a systematic review**

# **Supplementary – Search Strategies: Medline, Embase, APA PsycInfo, Web of Science & Grey Literature**

## **Database:** Ovid MEDLINE(R) ALL <1946 to June 24, 2022>

Search incorporates RCT filter: Cochrane Highly Sensitive Search Strategy for identifying randomized trials in MEDLINE: sensitivity-maximizing version (Lefebvre et al., 2022).

Lefebvre, C., Glanville, J., Briscoe, S., Featherstone, R., Littlewood, A., Marshall, C., Metzendorf, M.-I., Noel-Storr, A., Paynter, R., Rader, T., Thomas, J., Wieland, L. S. (2022) Technical Supplement to Chapter 4: Searching for and selecting studies. In J. P. T. Higgins, J. Thomas, J. Chandler, M. S. Cumpston, T. Li, M. J. Page, V. A. Welch (Eds), *Cochrane Handbook for Systematic Reviews of Interventions Version 6.3 (updated February 2022).* <https://training.cochrane.org/handbook>

| **#** | **Query** | **Results from 27 Jun 2022** |
| --- | --- | --- |
| 1 | (autis* or asperger* or Kanner* or ASD or ASC or childhood disintegrative disorder or (pervasive and development* and disorder*) or PDD or PDD-NOS or rett or retts or RTT).mp. or exp Autism Spectrum Disorder/ or exp Autistic Disorder/ or exp Asperger Syndrome/ or exp Child Development Disorders, Pervasive/ or exp Rett Syndrome/ | 91,971 |
| 2 | (mental health or mental illness or mental illnesses or mental wellbeing or psychological wellbeing or mental well-being or psychological well-being or mental disorder* or mental condition*).mp. [mp=title, abstract, original title, name of substance word, subject heading word, floating sub-heading word, keyword heading word, organism supplementary concept word, protocol supplementary concept word, rare disease supplementary concept word, unique identifier, synonyms] | 427,108 |
| 3 | (adult* or late life or later life or middle age* or mid-life or older people or older person* or geriatric* or elderly or older m?n or older wom?n).mp. [mp=title, abstract, original title, name of substance word, subject heading word, floating sub-heading word, keyword heading word, organism supplementary concept word, protocol supplementary concept word, rare disease supplementary concept word, unique identifier, synonyms] | 8,092,928 |
| 4 | randomized controlled trial.pt. | 571,576 |
| 5 | controlled clinical trial.pt. | 94,918 |
| 6 | randomized.ab. | 566,219 |
| 7 | placebo.ab. | 229,426 |
| 8 | drug therapy.fs. | 2,504,393 |
| 9 | randomly.ab. | 385,366 |
| 10 | trial.ab. | 605,650 |
| 11 | groups.ab. | 2,370,523 |
| 12 | 4 or 5 or 6 or 7 or 8 or 9 or 10 or 11 | 5,390,433 |
| 13 | exp animals/ not humans.sh. | 5,021,339 |
| 14 | 12 not 13 | 4,693,535 |
| 15 | exp Adult/ | 7,811,824 |
| 16 | Mental Health/ | 53,917 |
| 17 | mental disorders/ or exp anxiety disorders/ or exp "bipolar and related disorders"/ or exp "disruptive, impulse control, and conduct disorders"/ or exp dissociative disorders/ or exp elimination disorders/ or exp "feeding and eating disorders"/ or exp mood disorders/ or exp paraphilic disorders/ or exp personality disorders/ or exp "schizophrenia spectrum and other psychotic disorders"/ or exp sexual dysfunctions, psychological/ or exp sleep wake disorders/ or exp somatoform disorders/ or exp substance-related disorders/ or exp "trauma and stressor related disorders"/ | 1,023,569 |
| 18 | (anxiety disorder* or bipolar disorder* or disruptive disorder* or impulse control disorder* or conduct disorder* or dissociative disorder* or elimination disorder* or feeding disorder* or eating disorder* or mood disorder* or paraphilic disorder* or personality disorder* or schizophrenia or psychosis or psychotic disorder* or sexual dysfunction* or sleep wake disorder* or sleep disorder* or somatoform disorder* or substance-related disorder* or trauma disorder* or stressor related disorder*).mp. [mp=title, abstract, original title, name of substance word, subject heading word, floating sub-heading word, keyword heading word, organism supplementary concept word, protocol supplementary concept word, rare disease supplementary concept word, unique identifier, synonyms] | 556,321 |
| 19 | or/16-18 | 1,154,065 |
| 20 | 1 and (2 or 19) and 14 and (3 or 15) | 1,289 |

### Repeated search string <1946 to August 05, 2022>:

| 21 | limit 20 to dt=20220601-20220807 [between June 1st 2022 and 7th August 2022] | 11 |
| --- | --- | --- |

### Repeated search string <1946 to May 24, 2023>:

| 21 | limit 20 to dt=20220801-20230525 [between 1 August 2022 and 25 May 2023] | 76 |
| --- | --- | --- |

## **Database:** Embase <1974 to 2022 June 24> (Ovid)

Search incorporates the following RCT filter: Box 3.e Cochrane Highly Sensitive Search Strategy for identifying controlled trials in Embase: (2018 revision), (Lefebvre et al., 2019).

Lefebvre, C., Glanville, J., Briscoe, S., Littlewood, A., Marshall, C., Metzendorf, M.-I., Noel-Storr, A., Rader, T., Shokraneh, F., Thomas, J., Wieland, L. S. (2019) Technical Supplement to Chapter 4: Searching for and selecting studies. In J. P. T. Higgins, J. Thomas, J. Chandler, M. S. Cumpston, T. Li, M. J. Page, V. A. Welch (Eds), *Cochrane Handbook for Systematic Reviews of Interventions Version 6.* <https://training.cochrane.org/handbook>

| **#** | **Query** | **Results from 27 Jun 2022** |
| --- | --- | --- |
| 1 | Randomized controlled trial/ | 714,012 |
| 2 | Controlled clinical study/ | 465,861 |
| 3 | Random$.ti,ab. | 1,802,372 |
| 4 | randomization/ | 94,004 |
| 5 | intermethod comparison/ | 284,493 |
| 6 | placebo.ti,ab. | 342,399 |
| 7 | (compare or compared or comparison).ti. | 567,527 |
| 8 | ((evaluated or evaluate or evaluating or assessed or assess) and (compare or compared or comparing or comparison)).ab. | 2,517,471 |
| 9 | (open adj label).ti,ab. | 97,477 |
| 10 | ((double or single or doubly or singly) adj (blind or blinded or blindly)).ti,ab. | 257,607 |
| 11 | double blind procedure/ | 196,068 |
| 12 | parallel group$1.ti,ab. | 29,541 |
| 13 | (crossover or cross over).ti,ab. | 116,865 |
| 14 | ((assign$ or match or matched or allocation) adj5 (alternate or group$1 or intervention$1 or patient$1 or subject$1 or participant$1)).ti,ab. | 381,965 |
| 15 | (assigned or allocated).ti,ab. | 450,317 |
| 16 | (controlled adj7 (study or design or trial)).ti,ab. | 410,580 |
| 17 | (volunteer or volunteers).ti,ab. | 268,566 |
| 18 | human experiment/ | 580,175 |
| 19 | trial.ti. | 361,327 |
| 20 | or/1-19 | 5,804,767 |
| 21 | (random$ adj sampl$ adj7 ("cross section$" or questionnaire$1 or survey$ or database$1)).ti,ab. not (comparative study/ or controlled study/ or randomi?ed controlled.ti,ab. or randomly assigned.ti,ab.) | 9,042 |
| 22 | Cross-sectional study/ not (randomized controlled trial/ or controlled clinical study/ or controlled study/ or randomi?ed controlled.ti,ab. or control group$1.ti,ab.) | 313,896 |
| 23 | (((case adj control$) and random$) not randomi?ed controlled).ti,ab. | 19,927 |
| 24 | (Systematic review not (trial or study)).ti. | 213,265 |
| 25 | (nonrandom$ not random$).ti,ab. | 17,845 |
| 26 | "Random field$".ti,ab. | 2,727 |
| 27 | (random cluster adj3 sampl$).ti,ab. | 1,445 |
| 28 | (review.ab. and review.pt.) not trial.ti. | 1,003,940 |
| 29 | "we searched".ab. and (review.ti. or review.pt.) | 42,431 |
| 30 | "update review".ab. | 121 |
| 31 | (databases adj4 searched).ab. | 51,519 |
| 32 | (rat or rats or mouse or mice or swine or porcine or murine or sheep or lambs or pigs or piglets or rabbit or rabbits or cat or cats or dog or dogs or cattle or bovine or monkey or monkeys or trout or marmoset$1).ti. and animal experiment/ | 1,157,460 |
| 33 | Animal experiment/ not (human experiment/ or human/) | 2,428,712 |
| 34 | or/21-33 | 3,994,859 |
| 35 | 20 not 34 | 5,138,482 |
| 36 | (autis* or asperger* or Kanner* or ASD or ASC or childhood disintegrative disorder or (pervasive and development* and disorder*) or PDD or PDD-NOS or rett or retts or RTT).mp. [mp=title, abstract, heading word, drug trade name, original title, device manufacturer, drug manufacturer, device trade name, keyword heading word, floating subheading word, candidate term word] | 134,936 |
| 37 | (mental health or mental illness or mental illnesses or mental wellbeing or psychological wellbeing or mental well-being or psychological well-being or mental disorder* or mental condition*).mp. [mp=title, abstract, heading word, drug trade name, original title, device manufacturer, drug manufacturer, device trade name, keyword heading word, floating subheading word, candidate term word] | 464,790 |
| 38 | (adult* or late life or later life or middle age* or mid-life or older people or older person* or geriatric* or elderly or older m?n or older wom?n).mp. [mp=title, abstract, heading word, drug trade name, original title, device manufacturer, drug manufacturer, device trade name, keyword heading word, floating subheading word, candidate term word] | 10,009,047 |
| 39 | autism/ or asperger syndrome/ or childhood disintegrative disorder/ or "pervasive developmental disorder not otherwise specified"/ or rett syndrome/ | 84,367 |
| 40 | adult/ or exp aged/ or middle aged/ or young adult/ | 9,808,338 |
| 41 | mental health/ or psychological well-being/ | 196,492 |
| 42 | mental disease/ or exp addiction/ or adjustment disorder/ or exp anxiety disorder/ or exp behavior disorder/ or exp dissociative disorder/ or exp emotional disorder/ or exp mood disorder/ or exp personality disorder/ or exp psychosexual disorder/ or exp psychosis/ or exp psychosomatic disorder/ or exp psychotrauma/ or exp schizophrenia spectrum disorder/ | 1,782,073 |
| 43 | (mental disease* or addiction or adjustment disorder* or anxiety disorder* or behavio?r disorder* or dissociative disorder* or emotional disorder* or mood disorder* or personality disorder* or psychosexual disorder* or psychosis or psychosomatic disorder* or psychotrauma or schizophrenia spectrum disorder*).mp. [mp=title, abstract, heading word, drug trade name, original title, device manufacturer, drug manufacturer, device trade name, keyword heading word, floating subheading word, candidate term word] | 741,367 |
| 44 | 35 and (36 or 39) and (37 or 41 or 42 or 43) and (38 or 40) | 2,754 |
| 45 | limit 44 to english language | 2,687 |

### Repeated search Embase <1974 to 2022 August 05>:

| 46 | limit 45 to dc=20220601-20220807 [between June 1st 2022 and 7th August 2022] | 86 |
| --- | --- | --- |

### Repeated search Embase <1974 to 2023 May 24>:

| 46 | \|  \| limit 45 to dc=20220801-20230525 [between 1 August 2022 and 25 May 2023] \| \| --- \| --- \| | 432 |
| --- | --- | --- | --- | --- |

## **Database:** APA PsycInfo <1806 to June Week 3 2022> (Ovid)

Search incorporates RCT filter from:

Eady, A. M., Wilczynski, N. L., & Haynes, R. B. (2008). PsycINFO search strategies identified methodologically sound therapy studies and review articles for use by clinicians and researchers. *Journal of clinical epidemiology*, *61*(1), 34–40. <https://doi.org/10.1016/j.jclinepi.2006.09.016>

| **#** | **Query** | **Results from 27 Jun 2022** |
| --- | --- | --- |
| 1 | (autis* or asperger* or Kanner* or ASD or ASC or childhood disintegrative disorder or (pervasive and development* and disorder*) or PDD or PDD-NOS or rett or retts or RTT).mp. | 70,249 |
| 2 | (mental health or mental illness or mental illnesses or mental wellbeing or psychological wellbeing or mental well-being or psychological well-being or mental disorder* or mental condition*).mp. [mp=title, abstract, heading word, table of contents, key concepts, original title, tests & measures, mesh word] | 430,619 |
| 3 | (adult* or late life or later life or middle age* or mid-life or older people or older person* or geriatric* or elderly or older m?n or older wom?n).mp. [mp=title, abstract, heading word, table of contents, key concepts, original title, tests & measures, mesh word] | 1,164,506 |
| 4 | double-blind.tw. | 24,336 |
| 5 | random: assigned.tw. | 39,698 |
| 6 | control.tw. | 486,162 |
| 7 | or/4-6 | 528,003 |
| 8 | exp autism spectrum disorders/ or rett syndrome/ | 51,603 |
| 9 | exp mental disorders/ or exp affective disorders/ or exp anxiety disorders/ or exp bipolar disorder/ or exp chronic mental illness/ or exp dissociative disorders/ or exp eating disorders/ or exp paraphilias/ or exp personality disorders/ or exp psychosis/ or exp serious mental illness/ or exp sleep wake disorders/ or exp somatoform disorders/ or exp "stress and trauma related disorders"/ or exp "substance related and addictive disorders"/ or exp thought disturbances/ | 936,116 |
| 10 | (affective disorder* or anxiety disorder*or bipolar disorder* or chronic mental illness* or dissociative disorder* or eating disorder* or paraphilia* or personality disorder* or psychosis or serious mental illness* or sleep wake disorder* or somatoform disorder* or "stress and trauma related disorders" or "substance related and addictive disorders" or thought disturbance*).mp. [mp=title, abstract, heading word, table of contents, key concepts, original title, tests & measures, mesh word] | 220,430 |
| 11 | (1 or 8) and (2 or 9 or 10) and 3 and 7 | 2,134 |
| 12 | limit 11 to english language | 2,080 |

### Repeated search <1806 to August Week 1 2022>:

| 12 | limit 11 to up=20220601-20220807 [between June 1st 2022 and 7th August 2022] | 20 |
| --- | --- | --- |

Note: English language limit was not employed for this repeated search. This was unintentional. The rest of the search strategy was identical to the initial search.

### Repeated search <1806 to May Week 3 2023>:

| 13 | limit 12 to up=20220801-20230525 [between 1 August 2022 and 25 May 2023] | 61 |
| --- | --- | --- |

## **Database:** Web of Science searched 27/06/2022

Search incorporates Web of Science RCT filter adapted from:

University of Alberta. (n.d.) *Systematic Reviews, Scoping Reviews, and Health Technology Assessments - Searching the Literature.* University of Alberta. Retrieved June 27, 2022, from <https://guides.library.ualberta.ca/c.php?g=248586&p=1655962>

Search results: **844**

Query link: <https://www.webofscience.com/wos/woscc/summary/969730b0-8b45-4e4b-b536-f7c2d45762fb-3e57cb33/relevance/1>

Added mesh terms as keywords from the other database strategies in an ‘all fields’ (ALL) via the advanced search function causes WOS to exceed maximum number of terms allowed in one field. Searched mesh terms in the following fields with Boolean operators present: Title(TI), Abstract(AB), Author Keywords (AK) and Keyword Plus (KP). Direct copy-paste from the other database search strategies, so some duplication of terms exists. Manual deduplication did not take place to prevent the potential loss of terms through human error.

**Strategy:**

(((((TS=(randomized OR randomized OR randomisation OR randomization OR placebo* OR (random* AND (allocat* OR assign*) ) OR (blind* AND (single OR double OR treble OR triple) )) NOT TS=(animal or animals or pisces or fish or fishes or catfish or catfishes or sheatfish or silurus or arius or heteropneustes or clarias or gariepinus or fathead minnow or fathead minnows or pimephales or promelas or cichlidae or trout or trouts or char or chars or salvelinus or salmo or oncorhynchus or guppy or guppies or millionfish or poecilia or goldfish or goldfishes or carassius or auratus or mullet or mullets or mugil or curema or shark or sharks or cod or cods or gadus or morhua or carp or carps or cyprinus or carpio or killifish or eel or eels or anguilla or zander or sander or lucioperca or stizostedion or turbot or turbots or psetta or flatfish or flatfishes or plaice or pleuronectes or platessa or tilapia or tilapias or oreochromis or sarotherodon or common sole or dover sole or solea or zebrafish or zebrafishes or danio or rerio or seabass or dicentrarchus or labrax or morone or lamprey or lampreys or petromyzon or pumpkinseed or pumpkinseeds or lepomis or gibbosus or herring or clupea or harengus or amphibia or amphibian or amphibians or anura or salientia or frog or frogs or rana or toad or toads or bufo or xenopus or laevis or bombina or epidalea or calamita or salamander or salamanders or newt or newts or triturus or reptilia or reptile or reptiles or bearded dragon or pogona or vitticeps or iguana or iguanas or lizard or lizards or anguis fragilis or turtle or turtles or snakes or snake or aves or bird or birds or quail or quails or coturnix or bobwhite or colinus or virginianus or poultry or poultries or fowl or fowls or chicken or chickens or gallus or zebra finch or taeniopygia or guttata or canary or canaries or serinus or canaria or parakeet or parakeets or grasskeet or parrot or parrots or psittacine or psittacines or shelduck or tadorna or goose or geese or branta or leucopsis or woodlark or lullula or flycatcher or ficedula or hypoleuca or dove or doves or geopelia or cuneata or duck or ducks or greylag or graylag or anser or harrier or circus pygargus or red knot or great knot or calidris or canutus or godwit or limosa or lapponica or meleagris or gallopavo or jackdaw or corvus or monedula or ruff or philomachus or pugnax or lapwing or peewit or plover or vanellus or swan or cygnus or columbianus or bewickii or gull or chroicocephalus or ridibundus or albifrons or great tit or parus or aythya or fuligula or streptopelia or risoria or spoonbill or platalea or leucorodia or blackbird or turdus or merula or blue tit or cyanistes or pigeon or pigeons or columba or pintail or anas or starling or sturnus or owl or athene noctua or pochard or ferina or cockatiel or nymphicus or hollandicus or skylark or alauda or tern or sterna or teal or crecca or oystercatcher or haematopus or ostralegus or shrew or shrews or sorex or araneus or crocidura or russula or european mole or talpa or chiroptera or bat or bats or eptesicus or serotinus or myotis or dasycneme or daubentonii or pipistrelle or pipistrellus or cat or cats or felis or catus or feline or dog or dogs or canis or canine or canines or otter or otters or lutra or badger or badgers or meles or fitchew or fitch or foumart or foulmart or ferrets or ferret or polecat or polecats or mustela or putorius or weasel or weasels or fox or foxes or vulpes or common seal or phoca or vitulina or grey seal or halichoerus or horse or horses or equus or equine or equidae or donkey or donkeys or mule or mules or pig or pigs or swine or swines or hog or hogs or boar or boars or porcine or piglet or piglets or sus or scrofa or llama or llamas or lama or glama or deer or deers or cervus or elaphus or cow or cows or bos taurus or bos indicus or bovine or bull or bulls or cattle or bison or bisons or sheep or sheeps or ovis aries or ovine or lamb or lambs or mouflon or mouflons or goat or goats or capra or caprine or chamois or rupicapra or leporidae or lagomorpha or lagomorph or rabbit or rabbits or oryctolagus or cuniculus or laprine or hares or lepus or rodentia or rodent or rodents or murinae or mouse or mice or mus or musculus or murine or woodmouse or apodemus or rat or rats or rattus or norvegicus or guinea pig or guinea pigs or cavia or porcellus or hamster or hamsters or mesocricetus or cricetulus or cricetus or gerbil or gerbils or jird or jirds or meriones or unguiculatus or jerboa or jerboas or jaculus or chinchilla or chinchillas or beaver or beavers or castor fiber or castor canadensis or sciuridae or squirrel or squirrels or sciurus or chipmunk or chipmunks or marmot or marmots or marmota or suslik or susliks or spermophilus or cynomys or cottonrat or cottonrats or sigmodon or vole or voles or microtus or myodes or glareolus or primate or primates or prosimian or prosimians or lemur or lemurs or lemuridae or loris or bush baby or bush babies or bushbaby or bushbabies or galago or galagos or anthropoidea or anthropoids or simian or simians or monkey or monkeys or marmoset or marmosets or callithrix or cebuella or tamarin or tamarins or saguinus or leontopithecus or squirrel monkey or squirrel monkeys or saimiri or night monkey or night monkeys or owl monkey or owl monkeys or douroucoulis or aotus or spider monkey or spider monkeys or ateles or baboon or baboons or papio or rhesus monkey or macaque or macaca or mulatta or cynomolgus or fascicularis or green monkey or green monkeys or chlorocebus or vervet or vervets or pygerythrus or hominoidea or ape or apes or hylobatidae or gibbon or gibbons or siamang or siamangs or nomascus or symphalangus or hominidae or orangutan or orangutans or pongo or chimpanzee or chimpanzees or pan troglodytes or bonobo or bonobos or pan paniscus or gorilla or gorillas or troglodytes))) AND ALL=(autis* or asperger* or Kanner* or ASD or ASC or childhood disintegrative disorder or (pervasive and development* and disorder*) or PDD or PDD-NOS or rett or retts or RTT)) AND ALL=(adult* or late life or later life or middle age* or mid-life or older people or older person* or geriatric* or elderly or older m?n or older wom?n))) AND ((ALL=(mental health or mental illness or mental illnesses or mental wellbeing or psychological wellbeing or mental well-being or psychological well-being or mental disorder* or mental condition*) OR TI=(affective disorder* or anxiety disorder*or bipolar disorder* or chronic mental illness* or dissociative disorder* or eating disorder* or paraphilia* or personality disorder* or psychosis or serious mental illness* or sleep wake disorder* or somatoform disorder* or "stress and trauma related disorders" or "substance related and addictive disorders" or thought disturbance* or mental disease* or addiction or adjustment disorder* or anxiety disorder* or behavio$r disorder* or dissociative disorder* or emotional disorder* or mood disorder* or personality disorder* or psychosexual disorder* or psychosis or psychosomatic disorder* or psychotrauma or schizophrenia spectrum disorder* or anxiety disorder* or bipolar disorder* or disruptive disorder* or impulse control disorder* or conduct disorder* or dissociative disorder* or elimination disorder* or feeding disorder* or eating disorder* or mood disorder* or paraphilic disorder* or personality disorder* or schizophrenia or psychosis or psychotic disorder* or sexual dysfunction* or sleep wake disorder* or sleep disorder* or somatoform disorder* or substance-related disorder* or trauma disorder* or stressor related disorder*) OR AB=(affective disorder* or anxiety disorder*or bipolar disorder* or chronic mental illness* or dissociative disorder* or eating disorder* or paraphilia* or personality disorder* or psychosis or serious mental illness* or sleep wake disorder* or somatoform disorder* or "stress and trauma related disorders" or "substance related and addictive disorders" or thought disturbance* or mental disease* or addiction or adjustment disorder* or anxiety disorder* or behavio$r disorder* or dissociative disorder* or emotional disorder* or mood disorder* or personality disorder* or psychosexual disorder* or psychosis or psychosomatic disorder* or psychotrauma or schizophrenia spectrum disorder* or anxiety disorder* or bipolar disorder* or disruptive disorder* or impulse control disorder* or conduct disorder* or dissociative disorder* or elimination disorder* or feeding disorder* or eating disorder* or mood disorder* or paraphilic disorder* or personality disorder* or schizophrenia or psychosis or psychotic disorder* or sexual dysfunction* or sleep wake disorder* or sleep disorder* or somatoform disorder* or substance-related disorder* or trauma disorder* or stressor related disorder*) OR AK=(affective disorder* or anxiety disorder*or bipolar disorder* or chronic mental illness* or dissociative disorder* or eating disorder* or paraphilia* or personality disorder* or psychosis or serious mental illness* or sleep wake disorder* or somatoform disorder* or "stress and trauma related disorders" or "substance related and addictive disorders" or thought disturbance* or mental disease* or addiction or adjustment disorder* or anxiety disorder* or behavio$r disorder* or dissociative disorder* or emotional disorder* or mood disorder* or personality disorder* or psychosexual disorder* or psychosis or psychosomatic disorder* or psychotrauma or schizophrenia spectrum disorder* or anxiety disorder* or bipolar disorder* or disruptive disorder* or impulse control disorder* or conduct disorder* or dissociative disorder* or elimination disorder* or feeding disorder* or eating disorder* or mood disorder* or paraphilic disorder* or personality disorder* or schizophrenia or psychosis or psychotic disorder* or sexual dysfunction* or sleep wake disorder* or sleep disorder* or somatoform disorder* or substance-related disorder* or trauma disorder* or stressor related disorder*) OR KP = (affective disorder* or anxiety disorder*or bipolar disorder* or chronic mental illness* or dissociative disorder* or eating disorder* or paraphilia* or personality disorder* or psychosis or serious mental illness* or sleep wake disorder* or somatoform disorder* or "stress and trauma related disorders" or "substance related and addictive disorders" or thought disturbance* or mental disease* or addiction or adjustment disorder* or anxiety disorder* or behavio$r disorder or dissociative disorder* or emotional disorder* or mood disorder* or personality disorder* or psychosexual disorder* or psychosis or psychosomatic disorder* or psychotrauma or schizophrenia spectrum disorder* or anxiety disorder* or bipolar disorder* or disruptive disorder* or impulse control disorder* or conduct disorder* or dissociative disorder* or elimination disorder* or feeding disorder* or eating disorder* or mood disorder* or paraphilic disorder* or personality disorder* or schizophrenia or psychosis or psychotic disorder* or sexual dysfunction* or sleep wake disorder* or sleep disorder* or somatoform disorder* or substance-related disorder* or trauma disorder* or stressor related disorder*)))

### Repeated search added the following to the end of the above query string:

AND DOP=(2022-06-01/2022-08-07)

Search retrieved a further 10 records.

### Second repeated search added the following to the end of the initial query string:

AND DOP=(2022-08-01/2023-05-25)

Search retrieved a further 70 records.

## **Database:** APA PsycExtra <1908 to June 13, 2022>

Same strategy used as APA PsycInfo search, including Eady et al. (2008) RCT filter.

Eady, A. M., Wilczynski, N. L., & Haynes, R. B. (2008). PsycINFO search strategies identified methodologically sound therapy studies and review articles for use by clinicians and researchers. *Journal of clinical epidemiology*, *61*(1), 34–40. <https://doi.org/10.1016/j.jclinepi.2006.09.016>

| **#** | **Query** | **Results from 27 Jun 2022** |
| --- | --- | --- |
| 1 | (autis* or asperger* or Kanner* or ASD or ASC or childhood disintegrative disorder or (pervasive and development* and disorder*) or PDD or PDD-NOS or rett or retts or RTT).mp. | 1,896 |
| 2 | (mental health or mental illness or mental illnesses or mental wellbeing or psychological wellbeing or mental well-being or psychological well-being or mental disorder* or mental condition*).mp. [mp=title, abstract, heading word, keywords] | 27,617 |
| 3 | (adult* or late life or later life or middle age* or mid-life or older people or older person* or geriatric* or elderly or older m?n or older wom?n).mp. [mp=title, abstract, heading word, keywords] | 20,657 |
| 4 | double-blind.tw. | 196 |
| 5 | random: assigned.tw. | 1,471 |
| 6 | control.tw. | 16,942 |
| 7 | or/4-6 | 18,031 |
| 8 | exp autism spectrum disorders/ or rett syndrome/ | 1,485 |
| 9 | exp mental disorders/ or exp affective disorders/ or exp anxiety disorders/ or exp bipolar disorder/ or exp chronic mental illness/ or exp dissociative disorders/ or exp eating disorders/ or exp paraphilias/ or exp personality disorders/ or exp psychosis/ or exp serious mental illness/ or exp sleep wake disorders/ or exp somatoform disorders/ or exp "stress and trauma related disorders"/ or exp "substance related and addictive disorders"/ or exp thought disturbances/ | 38,264 |
| 10 | (affective disorder* or anxiety disorder*or bipolar disorder* or chronic mental illness* or dissociative disorder* or eating disorder* or paraphilia* or personality disorder* or psychosis or serious mental illness* or sleep wake disorder* or somatoform disorder* or "stress and trauma related disorders" or "substance related and addictive disorders" or thought disturbance*).mp. [mp=title, abstract, heading word, keywords] | 5,732 |
| 11 | (1 or 8) and (2 or 9 or 10) and 3 and 7 | 18 |
| 12 | limit 11 to english language | 18 |

### Repeated search string <1908 to July 11, 2022>:

| 12 | limit 11 to up=20220601-20220807 [between June 1st 2022 and 7th August 2022] | 1 |
| --- | --- | --- |

Note: English language limit was not employed for repeated search. This was unintentional. The rest of the search strategy was identical to the initial search.

### Repeated search string <1806 to May Week 3 2023>:

| 13 | limit 12 to up=20220801-20230525 [between 1 August 2022 and 25 May 2023] | 2 |
| --- | --- | --- |

## **ClinicalTrials.gov** Searched 27/06/2022

**96** Studies found for: **Interventional Studies | Autism | Adult, Older Adult | Phase 2, 3, 4**

### Repeated searches:

No Studies found for: **Studies With Results | Interventional Studies | Autism | Adult, Older Adult | Phase 2, 3, 4 | First posted from 06/01/2022 to 08/07/2022**

No Studies found for: **Studies With Results | Interventional Studies | Autism | Adult, Older Adult | Phase 2, 3, 4 | First posted from 08/01/2022 to 05/25/2023**

## **WHO International Clinical Trials Registry Platform (ICTRP)*** Searched 27/06/2022

ICTRP advanced search would not allow XML exports. Tried on multiple browsers (Microsoft Edge, Google Chrome, Firefox). Performed basic search for **‘autism’ restricted to trial phases 2-4, “with results”** only. Exported as XML file.

**82** records with results

### Repeated search, date of registration between 1 June 2022 and 7 August 2022:

“0 records for 0 trials found!”

### Repeated search, date of registration between 1 August 2022 and 25 May 2023:

“0 records for 0 trials found!”

*A website error was encountered with ICTRP whereby records could not be retrieved unless the search was restricted to trials “with results”. However, trials were only eligible for inclusion in this review where they contained results for autistic adults, so this was deemed acceptable.

# **Supplementary – Title & Abstract Screening Form**

**Q4:** Are the autistic participants **adults**, and are they considered separately?

**Q3:** Are some or all participants **autistic**/have a diagnosis of **autism** spectrum disorder?

**YES / not sure**

**NO**

The intervention being trialled is not targeting mental health problems. But, include studies targeting behavioural problems if participants have an intellectual disability.

**YES / not sure**

**Q1:** Is the study a randomised controlled trial (**RCT)**?

**NO**

The paper is a review/case study/cross-sectional/opinion piece/quasi-experimental/
conference proceedings
/letter/etc.

**Q2:** Is the intervention seeking to treat/prevent/ manage a **mental health problem?**

**NO**

The participants are not stated as being autistic or having a diagnosis of autism spectrum disorder (ASD), Aspergers, or other recognised diagnostic terms considered to be ASDs.

**YES / not sure**

**NO**

All participants are under the age of 18 or those aged 18 and above are within a child/adolescent group which includes youths aged 17 and under.

**YES / not sure**

**INCLUDE FOR NEXT STAGE**

# **Supplementary – Full-Text Screening Form**

**2^nd^ Stage/Full-text Screening of Search Results**

| **StudyID:** |
| --- |
| **Reviewer:** |
| **Date screened:** |

Please complete the questions below the line before giving a final decision. If you respond ‘no’ to a question, you can stop screening that study and pick ‘no’ as the final decision.

**Final Decision:**

Yes, include

No, exclude  *State relevant question number(s):*

Unsure  *State relevant question number(s):*

**Q1. Is the study a randomised controlled trial (RCT?)**

*Whereby an intervention is being trialled and participants from the population of interest have been randomly allocated to either an intervention group(s) or control/comparison group. RCTs can be open or blinded in multiple ways (e.g., single-blind, double-blind, quadruple-blind.)*

Yes

No

Unsure

If the response was ‘unsure’, please explain here:

**Q2. Is the intervention being delivered to individuals stated as being autistic or having a diagnosis of autism spectrum disorder under any recognised diagnostic terms?**

*Recognised diagnostic terms include: Autism Spectrum Disorder (ASD), Asperger’s Syndrome, Pervasive Developmental Disorder (PDD), Pervasive Developmental Disorder – Not Otherwise Specified, (PDD-NOS), Kanner’s Syndrome and Rett Syndrome.*

*Exclude if the only means of identification are autistic traits or self-diagnosis.*

Yes

No

Unsure

If the response was ‘unsure’, please explain here:

**Q3. Are the autistic individuals aged 18 years or above? And are their outcomes reported separately from participants under the age of 18?**

*Mixed-age participant groups where data from autistic adults cannot be isolated for extraction should be excluded.*

Yes

No

Unsure

If the response was ‘unsure’, please explain here:

**Q4. Is the RCT measuring mental health problems of participants, following a mental health intervention?**

*Mental health interventions are pharmacological, non-pharmacological, or combinations of pharmacological and non-pharmacological interventions that aim to prevent, treat or manage mental health problems and use standardised outcome measures specific to mental health problems.*

*If autistic participants are reported as having an intellectual disability or having an IQ <70, studies measuring behavioural problems which may indicate mental health problems should be included. E.g., self-injurious behaviour, aggression, and irritability.*

Yes

No

Unsure

If the response was ‘unsure’, please explain here:

# **Supplementary – Quality of Life Measures Checklist**

| Measure Name | Systematic Review Source | StudyID: |
| --- | --- | --- |
| AQLQ-M | Pequeno et al., 2020 |  |
| AQoL-4D | Pequeno et al., 2020 |  |
| Asthma Quality of Life Questionnaire (AQLQ) | Haraldstad et al., 2019 |  |
| Cantrills ladder | Haraldstad et al., 2019 |  |
| CAS 20 | Haraldstad et al., 2019 |  |
| CASP-12 | Haraldstad et al., 2019 |  |
| CASP-16 | Pequeno et al., 2020 |  |
| CDC-HRQoL-14 | Pequeno et al., 2020 |  |
| CDC-HRQoL-4 | Pequeno et al., 2020 |  |
| Celiac dietary, CD quality of life | Haraldstad et al., 2019 |  |
| Comprehensive Quality of Life Questionnaire (ComQOL) | Ayres et al., 2017 |  |
| CQoLC-K | Pequeno et al., 2020 |  |
| Dartmount coopertive functional assessment charts (COOP) | Haraldstad et al., 2019 |  |
| Dermatology Life Quality score, (DLQ1) | Haraldstad et al., 2019 |  |
| DLQI | Pequeno et al., 2020 |  |
| EORTC-QLQ-C30 | Pequeno et al., 2020 |  |
| Epilepsy and Learning Disabilities Quality of Life Scale (ELDQOL) | Haraldstad et al., 2019 |  |
| EQ-5D | Pequeno et al., 2020 |  |
| EQ-VAS | Pequeno et al., 2020 |  |
| EUROHIS-QoL 8-item | Pequeno et al., 2020 |  |
| FACT-L | Haraldstad et al., 2019 |  |
| FertiQOL | Haraldstad et al., 2019 |  |
| FLQA-d | Pequeno et al., 2020 |  |
| Food Allergy Quality of Life Parental Burden | Haraldstad et al., 2019 |  |
| Gastrointestinal QOL index (GIQLI) | Haraldstad et al., 2019 |  |
| Glaucoma-specific preference-based HRQOL instrument | Haraldstad et al., 2019 |  |
| Incontinence Impact Questionnaire Short Form (31Q, IIQ-7) | Haraldstad et al., 2019 |  |
| Inflam. Bowel Disease Questionnaire (IBDQ) | Haraldstad et al., 2019 |  |
| Kansas City Cardiomyopathy Questionnaire (KCCQ) | Haraldstad et al., 2019 |  |
| McGill QOL Questionnaire | Haraldstad et al., 2019 |  |
| Medical outcomes study short form health survey version 2 (SF-12 v.2) | Ayres et al., 2017 |  |
| Melasma QOL questionnaire | Haraldstad et al., 2019 |  |
| MG-QOL 15 | Haraldstad et al., 2019 |  |
| National Eye Institute Visual Functioning Questionnaire (NEI-VFQ-25) | Haraldstad et al., 2019 |  |
| NOSE nasal obstruction symptom evaluation | Haraldstad et al., 2019 |  |
| Novel QoL measures (QOL1 and QOL2) | Ayres et al., 2017 |  |
| Nutri- QOL | Haraldstad et al., 2019 |  |
| OHIP-14 | Pequeno et al., 2020 |  |
| OIDP | Pequeno et al., 2020 |  |
| Owestry dis index (ODI) | Haraldstad et al., 2019 |  |
| Patient Health Questionnaire-2 score (PHQ2) | Haraldstad et al., 2019 |  |
| Patient outcome measurement information system (PROMIS) | Haraldstad et al., 2019 |  |
| QLESQ-SF | Haraldstad et al., 2019 |  |
| QOL Alzheimer’s Disease Scale | Haraldstad et al., 2019 |  |
| QoL scale | Pequeno et al., 2020 |  |
| Quality of Life Inventory (QOLI) | Ayres et al., 2017 |  |
| Quality of Life Questionnaire (QoL-Q) | Ayres et al., 2017 |  |
| RTQ | Pequeno et al., 2020 |  |
| Satisfaction with Life Scale (SWLS) | Haraldstad et al., 2019 |  |
| Seattle Angina Questionnaire (SAQ-QOL) | Haraldstad et al., 2019 |  |
| Sexual Function Questionnaire-12 (PISQ-12) | Haraldstad et al., 2019 |  |
| SF-12 | Pequeno et al., 2020 |  |
| SF-8 | Pequeno et al., 2020 |  |
| Short Form Health Survey (SF-36) | Ayres et al., 2017 |  |
| SIBDQ | Pequeno et al., 2020 |  |
| Stoma-QOL Questionnaire | Haraldstad et al., 2019 |  |
| Stroke Specific QOL Scale | Haraldstad et al., 2019 |  |
| The Haemo-QOL Questionnaire | Haraldstad et al., 2019 |  |
| The Hand-Foot Skin Reaction QOL Questionnaire (HF-QOL-K) | Haraldstad et al., 2019 |  |
| The ten-item Lehman’s quality of life (QOL) measure | Haraldstad et al., 2019 |  |
| Visual Function/QoL | Pequeno et al., 2020 |  |
| World Health Organization Quality of Life (WHOQOL)-BREF | Ayres et al., 2017 |  |
| Other (please specify): | n/a |  |

Non-adult measures and duplicates were removed.

# **Supplementary – Results of CASP Randomised Controlled Trial Standard Checklist for All Included Reports**

| Questions | Braden et al.2022 | Chien et al.2021 | Coulter et al.2022 | Danforth et al.2018 | Gaigg et al.2020 | Hesselmark et al. 2014 | Horwood et al. 2021 | Lee et al.2022 | McDougle et al.1998 | McVey et al.2016 | Oh et al.2021 | Oswald et al.2018 | Pagni et al.2020 | Pagni et al.2023 | Pahnke et al.2022 | Quadt et al.2021 | Russell et al.2019 | Russell et al.2020 | Spek et al.2013 | Strydom et al.2020 | Wijker et al.2020 | Willemsen-Swinkels et al. 1995 |
| --- | --- | --- | --- | --- | --- | --- | --- | --- | --- | --- | --- | --- | --- | --- | --- | --- | --- | --- | --- | --- | --- | --- |
| 1. Did the study address a clearly focused research question? | **Y** | **Y** | **Y** | **Y** | **Y** | **Y** | **Y** | **Y** | **Y** | **Y** | **N** | **Y** | **Y** | **Y** | **Y** | **Y** | **Y** | **Y** | **Y** | **Y** | **Y** | **Y** |
| 2. Was the assignment of participants to the interventions randomized? | **Y** | **N** | **Y** | **?** | **N** | **N** | **Y** | **Y** | **Y** | **N** | **Y** | **N** | **Y** | **?** | **Y** | **Y** | **Y** | **Y** | **Y** | **Y** | **Y** | **?** |
| 3. Were all participants who entered the study accounted for at its conclusion? | **Y** | **Y** | **Y** | **Y** | **Y** | **Y** | **Y** | **Y** | **Y** | **Y** | **Y** | **Y** | **Y** | **Y** | **Y** | **Y** | **Y** | **Y** | **Y** | **?** | **Y** | **Y** |
| 4a. Were the participants ‘blind’ to intervention they were given? | **Y** | **N** | **N** | **Y** | **N** | **N** | **N** | **N** | **Y** | **N** | **N** | **N** | **?** | **Y** | **N** | **N** | **N** | **N** | **N** | **N** | **N** | **Y** |
| 4b. Were the investigators ‘blind’ to the intervention they were giving to participants? | **N** | **N** | **N** | **Y** | **N** | **N** | **?** | **?** | **Y** | **?** | **N** | **N** | **N** | **?** | **N** | **N** | **?** | **?** | **N** | **N** | **N** | **Y** |
| 4c. Were the people assessing/analysing outcome/s ‘blinded’? | **Y** | **?** | **N** | **Y** | **?** | **N** | **?** | **?** | **Y** | **?** | **N** | **N** | **?** | **Y** | **?** | **N** | **Y** | **Y** | **N** | **Y** | **Y** | **?** |
| 5. Were the study groups similar at the start of the randomised controlled trial? | **Y** | **Y** | **?** | **N** | **N** | **?** | **?** | **Y** | **Y** | **?** | **N** | **?** | **Y** | **Y** | **N** | **?** | **Y** | **Y** | **Y** | **N** | **?** | **?** |
| 6. Apart from the experimental intervention, did each study group receive the same level of care (that is, were they treated equally)? | **Y** | **N** | **?** | **Y** | **?** | **?** | **Y** | **Y** | **Y** | **Y** | **Y** | **?** | **Y** | **Y** | **Y** | **Y** | **Y** | **Y** | **Y** | **Y** | **Y** | **N** |
| 7. Were the effects of intervention reported comprehensively? | **Y** | **N** | **N** | **Y** | **N** | **Y** | **Y** | **?** | **N** | **Y** | **N** | **N** | **?** | **Y** | **Y** | **Y** | **?** | **?** | **?** | **Y** | **?** | **N** |
| 8. Was the precision of the estimate of the intervention or treatment effect reported? | **N** | **N** | **N** | **N** | **N** | **N** | **N** | **N** | **N** | **N** | **N** | **Y** | **N** | **N** | **N** | **Y** | **N** | **N** | **Y** | **Y** | **Y** | **N** |
| 9. Do the benefits of the experimental intervention outweigh the harms and costs? | **?** | **?** | **?** | **?** | **N** | **?** | **?** | **?** | **?** | **?** | **?** | **?** | **?** | **?** | **?** | **Y** | **?** | **?** | **?** | **N** | **?** | **N** |
| 10. Can the results be applied to your local population/in your context? | **N** | **?** | **?** | **?** | **?** | **N** | **N** | **?** | **?** | **?** | **N** | **?** | **N** | **N** | **N** | **?** | **?** | **?** | **N** | **N** | **N** | **N** |
| 11. Would the experimental intervention provide greater value to the people in your care than any of the existing interventions? | **?** | **?** | **?** | **?** | **?** | **?** | **?** | **Y** | **?** | **?** | **?** | **?** | **?** | **?** | **?** | **Y** | **?** | **?** | **?** | **?** | **?** | **N** |
| Author’s judgment of quality: Low (L), Medium (M), High (H) | **H** | **L** | **L** | **M** | **L** | **L** | **M** | **M** | **M** | **L** | **L** | **L** | **M** | **H** | **M** | **M** | **M** | **M** | **M** | **M** | **M** | **L** |
| Was the RCT statistically powered? Yes (Y), No (N), Not reported (?) | **N** | **?** | **N** | **N** | **N** | **N** | **N** | **N** | **?** | **Y** | **N** | **N** | **N** | **Y** | **N** | **Y** | **N** | **N** | **?** | **Y** | **N** | **?** |

Notes:

CASP ratings: Yes (Y), No (N) or Can’t Tell (?)

The final two rows are not part of the CASP but were added to facilitate analysis. Judgment was made according to whether screening questions were satisfied (1–3), overall number of criteria met, and whether criteria seven was satisfied, which was considered essential due to the large number of CASP considerations, all directly related to study results. Whether a study was preliminary or not was also taken into account. Studies not statistically powered included those stated to be exploratory, feasibility, pilot or preliminary in nature. Braden et al. (2022) and Pagni et al. (2020) are papers reporting on the same study. Horwood et al., (2021), Russell et al. (2019) and Russell et al. (2020) are papers reporting on the same study.
